# Levoglucosenone: Bio-Based Platform for Drug Discovery

**DOI:** 10.3389/fchem.2022.902239

**Published:** 2022-05-31

**Authors:** Jason E. Camp, Ben W. Greatrex

**Affiliations:** ^1^ Circa Sustainable Chemicals, York, United Kingdom; ^2^ School of Science and Technology, University of New England, Armidale, NSW, Australia

**Keywords:** levoglucosenone, bio-mass, platform chemical, chiral building block, drug discovery

## Abstract

Levoglucosone (LGO) is a bio-privileged molecule that can be produced on scale from waste biomass. This chiral building block has been converted via well-established chemical processes into previously difficult-to-synthesize building blocks such as enantiopure butenolides, dihydropyrans, substituted cyclopropanes, deoxy-sugars and ribonolactones. LGO is an excellent starting material for the synthesis of biologically active compounds, including those which have anti-cancer, anti-microbial or anti-inflammatory activity. This review will cover the conversion of LGO to biologically active compounds as well as provide future research directions related to this platform molecule.

## Introduction

**FIGURE 1:**
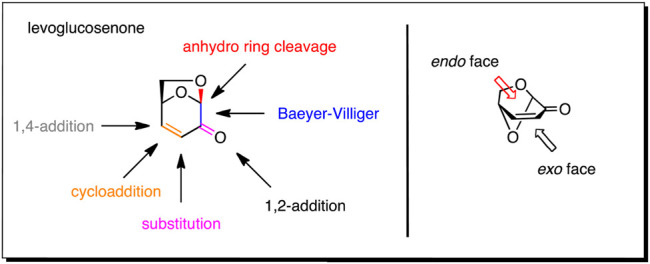
Reactions useful for derivitizing levoglucosenone.

Levoglucosenone (LGO, 1) is a chiral building block that is readily available from the pyrolysis of materials containing cellulose, including biomass waste such as wood chips and bagasse. Over the past 50 years, research into the chemistry of LGO has established the high degree of orthogonality in the reactive functional groups, and demonstrated the excellent stereochemical control offered by the bicyclic ring-system (Witczak and Tatsuta, 2002; Witczak, 2007; Sarotti et al., 2012a; Comba et al., 2018; Kühlborn et al., 2020). The chirality at C1 and C5 in LGO gives it advantages in stereoselective synthesis compared to achiral biomass derivatives such as furfural, while the reduced number of chiral centers simplifies its use compared to monosaccharides. Enantio-, stereo-, regio- and chemoselective reactions have been executed around the core ring-system. These include, 1,6-anhydro ring opening (Shafizadeh et al., 1979; Tagirov et al., 2015; Comba et al., 2016; Pedersen and Pedersen, 2021), Baeyer-Villiger oxidation (Koseki et al., 1990; Koseki et al., 1991; Teixeira et al., 2016; Bonneau et al., 2018; Diot-Neant et al., 2018), 1,2-addition (Shafizadeh and Chin, 1977; Tsypysheva et al., 2000; Giordano et al., 2012; Debsharma et al., 2019; Sharipov et al., 2019), α-substitution (Ward and Shafizadeh, 1981; Ledingham E. et al., 2017; Ledingham E. T. et al., 2017; Giri et al., 2017; Hughes et al., 2018; Liu et al., 2020), cycloaddition/cyclization (Yatsynich et al., 2003; Novikov et al., 2009; Faizullina et al., 2011; Samet et al., 2011; Sarotti et al., 2012b; Banwell et al., 2020; Fadlallah et al., 2020; Liu et al., 2020), and conjugate addition reactions (Shafizadeh et al., 1982; Essig, 1986; Samet et al., 1996; Trahanovsky et al., 2003) (Figure 1). These reactions have led to the controlled synthesis of a multitude of important biologically active motifs, including enantiopure butenolides, dihydropyrans, substituted cyclopropanes, deoxy-sugars and ribonolactones. The reactivity of the ketone and enone functionalities is influenced by the 1,6-anhydro bridge, which strongly biases reaction to occur from the less hindered *exo*-face of the molecule. Whilst the reactivity and potential of this molecule is well understood, it is only recently that it has been produced on industrial scale allowing its use as a chiral feedstock (Lawrence et al., 2012). This review will focus on the use of LGO as a starting material used for the synthesis of biologically active materials, specifically: known bioactive compounds, analogues of bioactives where the intact LGO ring-system has been incorporated into the final structure as a bioisostere, and novel materials. In addition, this review will briefly highlight how LGO derivatives can intercept routes to drugs currently in production.

### Synthesis of Known Biologically Active Molecules

**FIGURE 2:**
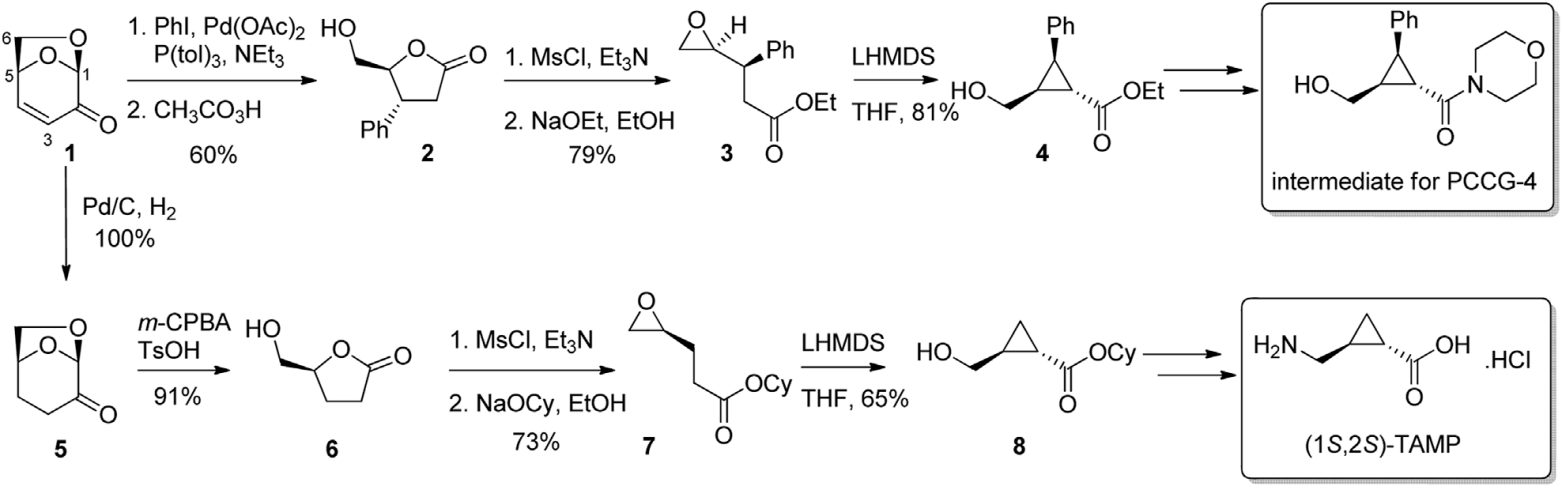
Preparation of bioactive cyclopropanes from LGO.

Enantiopure cyclopropanes are found in a variety of existing biologically active molecules, and are favoured for their rigidity and relative stability under biological conditions. There are a number of approaches for the preparation of cyclopropanes from LGO including the direct cyclopropanation of the alkene of either LGO or one of its derivatives (Samet et al., 2007; Ledingham E. et al., 2017), reactions of malonates with the 3-iododerivative of LGO (Valeev et al., 1999), or by transformation of LGO into a suitably reactive species for cyclization. Using this last approach, Stockton and Greatrex (2016) converted LGO into substituted butyrolactones via either 1) reductive cross-coupling and Baeyer-Villiger oxidation, or 2) reduction then Baeyer-Villiger oxidation (Figure 2). Lactone 2 and 6 were then converted to epoxides 3 and 7 using standard protocols. Finally, treatment of the epoxyesters 3 or 7 with lithium hexamethyldisilazide (LiHMDS) in tetrahydrofuran (THF) gave cyclopropanes 4 and 8, which following elaboration of functional groups led to an intermediate used for the synthesis of the selective glutamate receptor antagonist PCCG-4 and GABA_c_ receptor agonist (1*S*, 2*S*)-TAMP, respectively.

**FIGURE 3:**
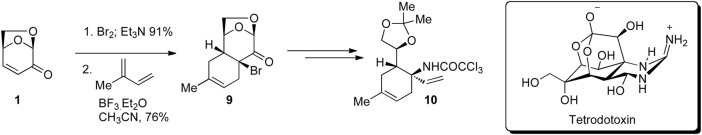
Synthesis of tetrodotoxin key intermediate from LGO.

Tetrodotoxin is the principal toxin of the pufferfish that acts through specific inhibition of sodium ion influx through excitable membranes. In a series of publications, Isobe and co-workers reported the first enantioselective total synthesis of this potent biologically active compound using LGO as a key building block (Figure 3) (Bamba et al., 1996; Urabe et al., 2006; Nishikawa and Isobe, 2013). This example demonstrates the utility and reaction control possible using LGO, whereby a one-pot two step C3 bromination of LGO followed by Diels–Alder cycloaddition afforded tricycle 9 as a single regio- and diastereomeric isomer. This transformation set key stereocenters that were then relayed into the final natural product tetrodotoxin via key intermediate 10.

**FIGURE 4:**
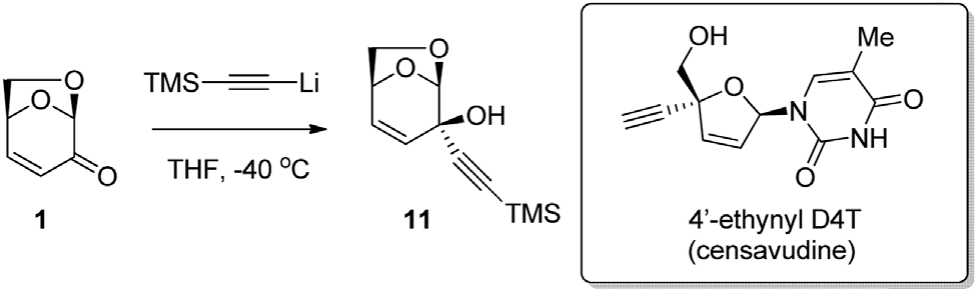
Key synthetic step towards censavudine from LGO.

Oncolys Bipharma used the reaction of LGO with TMS-acetylide to give alkyne-alcohol 11 as a result of selective 1,2-addition of the nucleophile to the ketone (Figure 4) (Nagai et al., 2015). The bicyclic alcohol 11 was then converted to 4′-ethynyl D4T (censavudine), which is a stavudine analogue with decreased cytotoxicity. This route via LGO allowed for the synthesis of the active substance in a simpler way, at lower cost and in larger quantities than previous routes. Censavudine has been investigated as a novel drug therapy for human immunodeficiency (HIV), with greater activity against the less common HIV-2 variant (Smith et al., 2015).

### Levoglucosenone in Analogue Synthesis or as a Bioisostere

**FIGURE 5:**
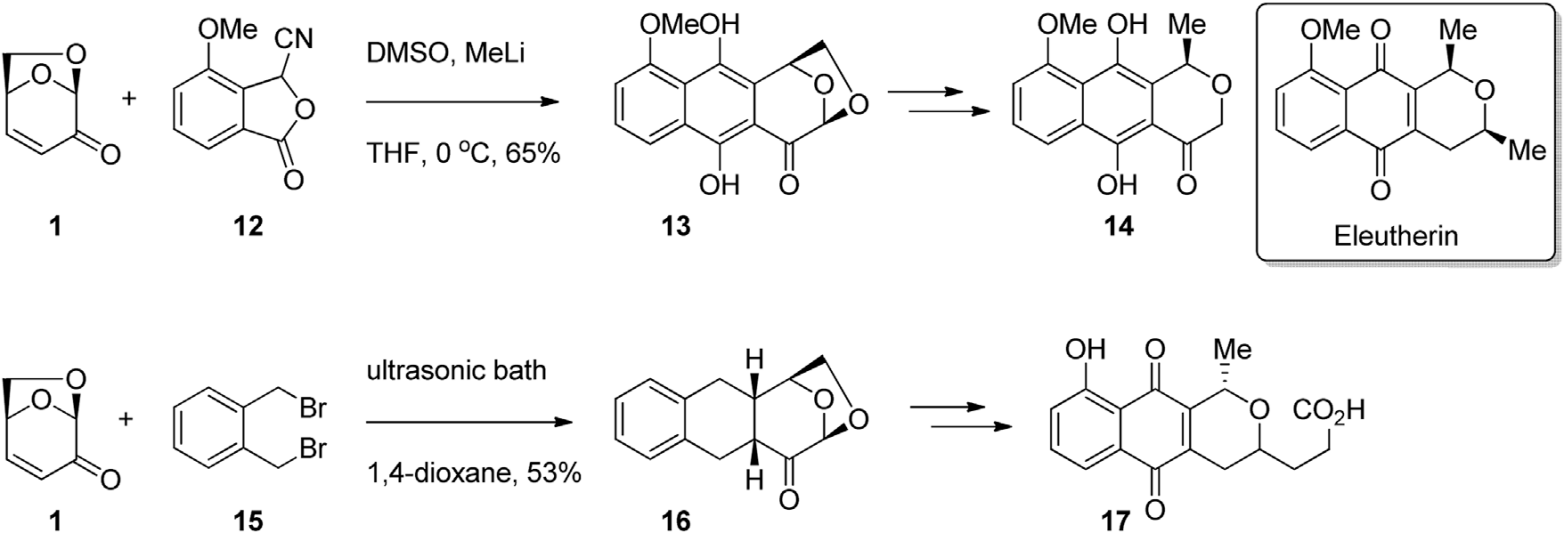
Pyranonaphthoquinones prepared from LGO.

In addition to the synthesis of known biologically active compounds, LGO has been used extensively in the synthesis of analogues of bioactives. For example, many substituted napthoquinone compounds are known to have antibiotic activity, such as the natural product eleutherin isolated from *Eleutherine bulbosa* (Bianchi and Ceriotti, 1975). LGO has been used for the synthesis of a series of quinone derivatives, with the core of the LGO converted into a tetrahydropyran or dihydropyranone ring. Freskos and Swenton (1985) performed a cycloaddition reaction between LGO and benzofuranone 12 to afford tetracycle 13 in moderate yield (Figure 5). Opening of the acetal ring and deoxygenation afforded napthohydroquinone derivative 14 as a single enantiomer, while a later report from the same group gave the natural product hongkonin (Swenton et al., 1996). Chew et al. (1988) subjected LGO and dibromoxylene 15 to sonication to afford tetracycle 16 as a single enantiomer (Figure 5). Functional group modification gave pyranonaphthoquinone derivative 17.

**FIGURE 6:**
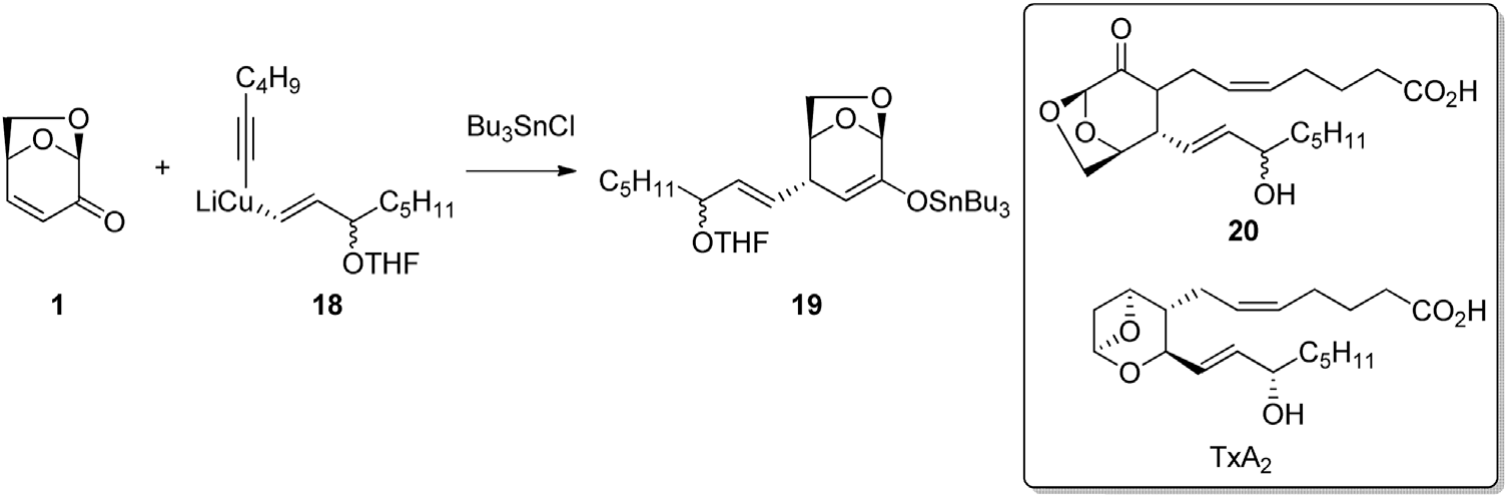
1,4-Additions of vinyl cuprates to LGO for the preparation of thromboxane analogues.

Conjugate addition to the enone functionality of LGO has also been used to synthesize biologically active derivatives of known compounds. Thromboxanes play a major role in blood clot formation (thrombosis) and are formed via the oxidation of arachidonic acid *in vivo*. Tolstikov and Tolstikov (2007) employed the LGO ring-system as a bioisostere for the cyclic ether motif found in thromboxane A2. The authors used the regio- and stereoselective addition of a mixed cuprate 18 to LGO to give the alkene addition product 19 as a key step in their synthesis of the thromboxane analogue (Figure 6). Alkylation of tin enolate 19 followed by deprotection afford the desired thromboxane analogue 20.

**FIGURE 7:**
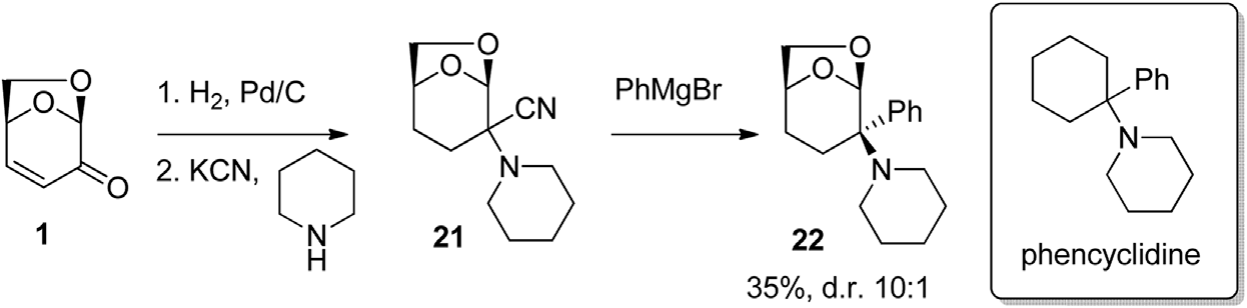
Preparation of phencyclidine analogues using LGO.

The use of the 6,8-dioxabicyclo [3.2.1]octane as a bioisostere for the cyclohexyl group was examined by Eiden et al. (1994) in their study of materials that bind to the *N*-methyl-d-aspartate receptor NMDA receptor complex (Figure 7). Starting with LGO, reduction and then Strecker reaction afforded the aminonitrile 21. Reactions of the aminonitrile 21 with aryl Grignard reagents gave a series of CNS-active arylamines including 22, which was shown to possess low micromolar activity at the NMDA receptor.

### Synthesis of Novel Bioactive Materials

**FIGURE 8:**
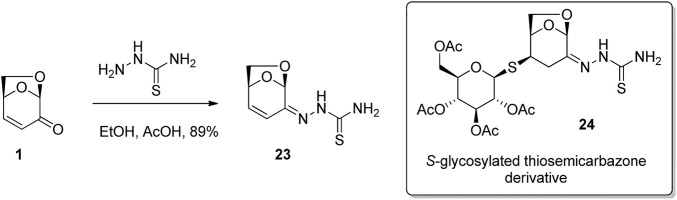
Anticancer thioglycosides prepared from LGO.

Due to the highly functionalized and rigid nature of LGO, as well as its well understood reactivity, it has been used extensively as a building block for the synthesis of novel biologically active compounds. 1,2-Addition to the carbonyl of LGO was used by Czubatka-Bienkowska *et al.* (2017) for the preparation of *S*-glycosylated thiosemicarbazone derivates, a class of molecules that have shown potential medical applications as antiviral, antibacterial and anticancer drugs. Thus, condensation of the thiosemicarbazide with LGO in acetic acid in ethanol afforded the desired adduct 23 in good yield (Figure 8). Subsequent conjugate addition of the 1-thioglucose derivative to thiosemicarbazone 23 gave *S*-glycosylated thiosemicarbazone 24. Testing of the compound library for *in vitro* anticancer activity showed significant activity against A2780 cancer cell line via induction of DNA damage, though this effect is not associated with apoptosis or oxidative stress induction.

**FIGURE 9:**
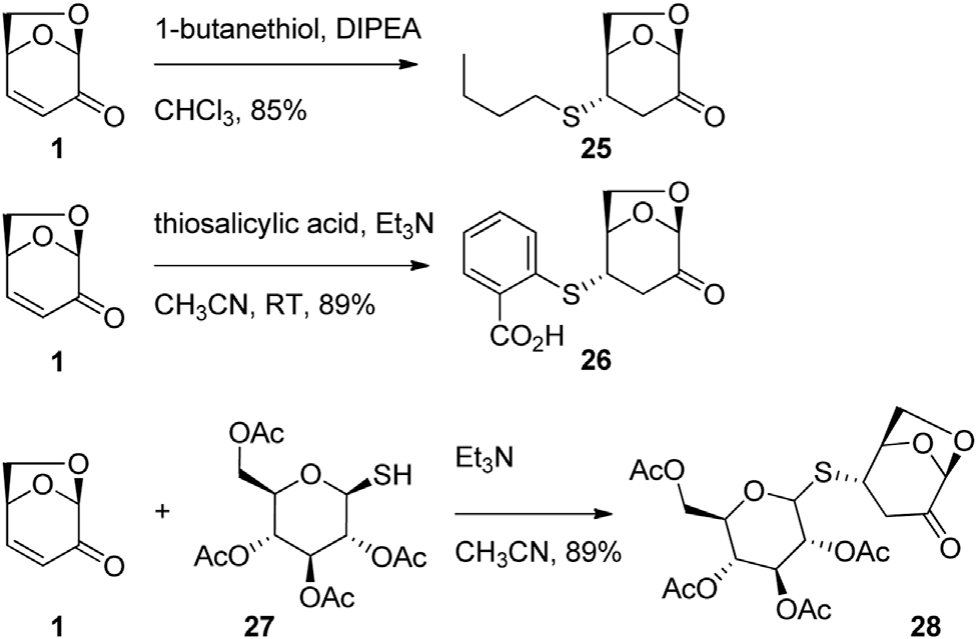
Anticancer compounds through conjugate addition of thiols to LGO.

The 1,4-addition of thiols to LGO has been used extensively for the synthesis of biologically active levoglucosenone derivatives. Giri *et. al.* (2016) used the reaction of 1-hexanethiol with LGO in the presence of a base to afford adduct 25 in good yield (Figure 9). The series of thio-derivatives synthesized in this way showed activity against hepatocarcinoma cell lines and also illuminated the key role of the carbonyl functionality to exert biological activity. Similarly, Witczak et al. (2014) demonstrated that substituted thiophenols could be efficiently added to LGO in the presence of triethylamine to give thio-acid 26 (Figure 9). In the same report, 1-thiosugars were shown to be competent nucleophiles for conjugate addition into LGO. For example, addition of protected 1-thioglucose 27 to LGO in the presence of triethylamine afford the desired adduct 28 in good yield (Figure 9). Importantly, this series of functional CARB-pharmacophores demonstrated cytotoxicity and apoptosis against human cancel cell lines (A549, LoVo, MCF-7 and HeLa). The thio-sugar motif was shown to be a promising construct for the development of novel antineoplastic drugs.

**FIGURE 10:**
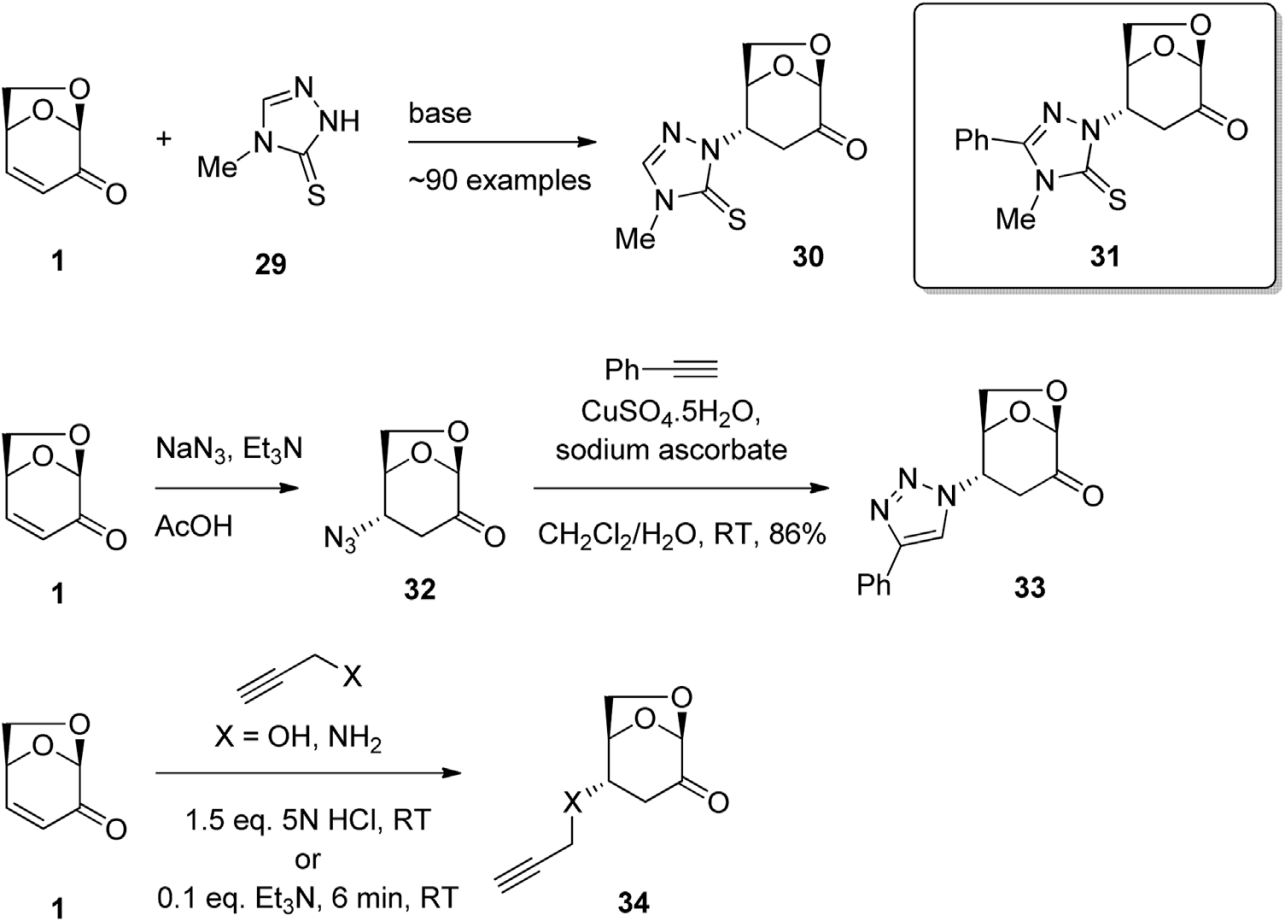
Anticancer derivatives of LGO via aza-Michael addition reactions.

Nitrogen and oxygen nucleophiles have also been shown to undergo conjugate addition to LGO and a number of biologically active derivatives have been formed using this chemistry. For example, Westman et al. (2007) have patented the synthesis of LGO derivatives for the treatment of disorders such as cancer, autoimmune diseases and heart diseases (Figure 10). The reaction of LGO with triazole derivative 29 in the presence of base afford the desired 1,4-addition adduct 30 as a single enantiomer. The approximately 90 derivatives were tested in a range of assays, including in human H1299 lung carcinoma cells lacking p53 expression and in H1299 His175 cells that carry tetracycline-regulated mutant p53 constructs. From these studies, a group of LGO derivatives were identified including 31 that showed promise in the treatment of disorders in which a malfunctioning p53 pathway could be involved. In a similar synthetic approach, Sarotti and co-workers used the reaction of sodium azide in acetic acid with LGO in the presence of triethylamine to give β-azidoketone 32 (Figure 10) (Tsai et al., 2018). A subsequent click reaction with phenyl acetylene performed without isolation of the β-azidoketone 32 afforded triazole 33. Application of the methodology to a diverse series of acetylenes gave triazole products, which showed satisfactory antitumor activity when evaluated against TNBC cancer cell lines. Primary amines have also been shown to be good nucleophiles for addition to the enone functionality of LGO. For example, further work by Sarotti and co-workers showed that the reaction of propargyl amine to LGO in the presence of base gave addition adduct 34 (Figure 10) (Tsai et al., 2020). Testing of this chain extended series on MDA-MB-231 cells, specifically endogenous mutant p53 knock down (R280K), and by reintroducing p53 R280K in cells lacking p53 expression, anti-proliferative activities against lung and colon cancer cell lines were demonstrated. Further examples of LGO derivatives with anticancer activity have been reported by the same group (Delbart et al., 2022).

**FIGURE 11:**
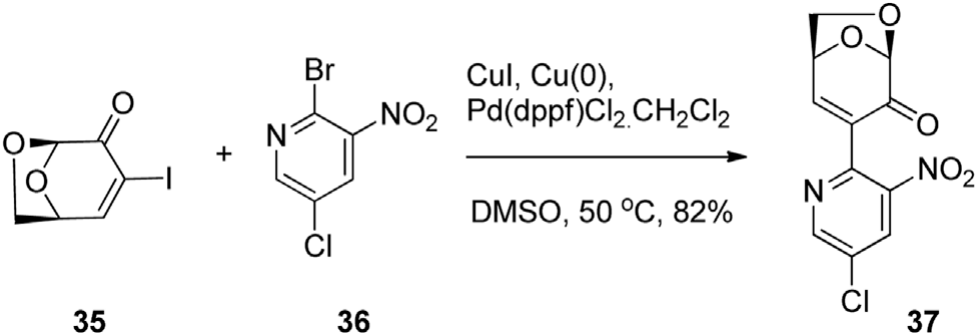
Antimicrobial and anticancer derivatives of LGO via cross-coupling reactions.

Carbon-Carbon bond formation via metal-mediated cross-couplings has also been used for the production of novel biologically active molecules based on LGO. Banwell and co-workers employed a palladium-catalyzed Ullmann cross-coupling between the C3 iodo derivative of LGO 35 and a variety of bromonitropyridines such as 36 affording a range of LGO derivatives (Figure 11) (Liu et al., 2020). It was found that the α-pyridinylated derivatives of type 37 had potent and selective antimicrobial and/or cytotoxic properties, whereas the azaindole derivatives were essentially inactive in all of the tests conducted.

**FIGURE 12:**
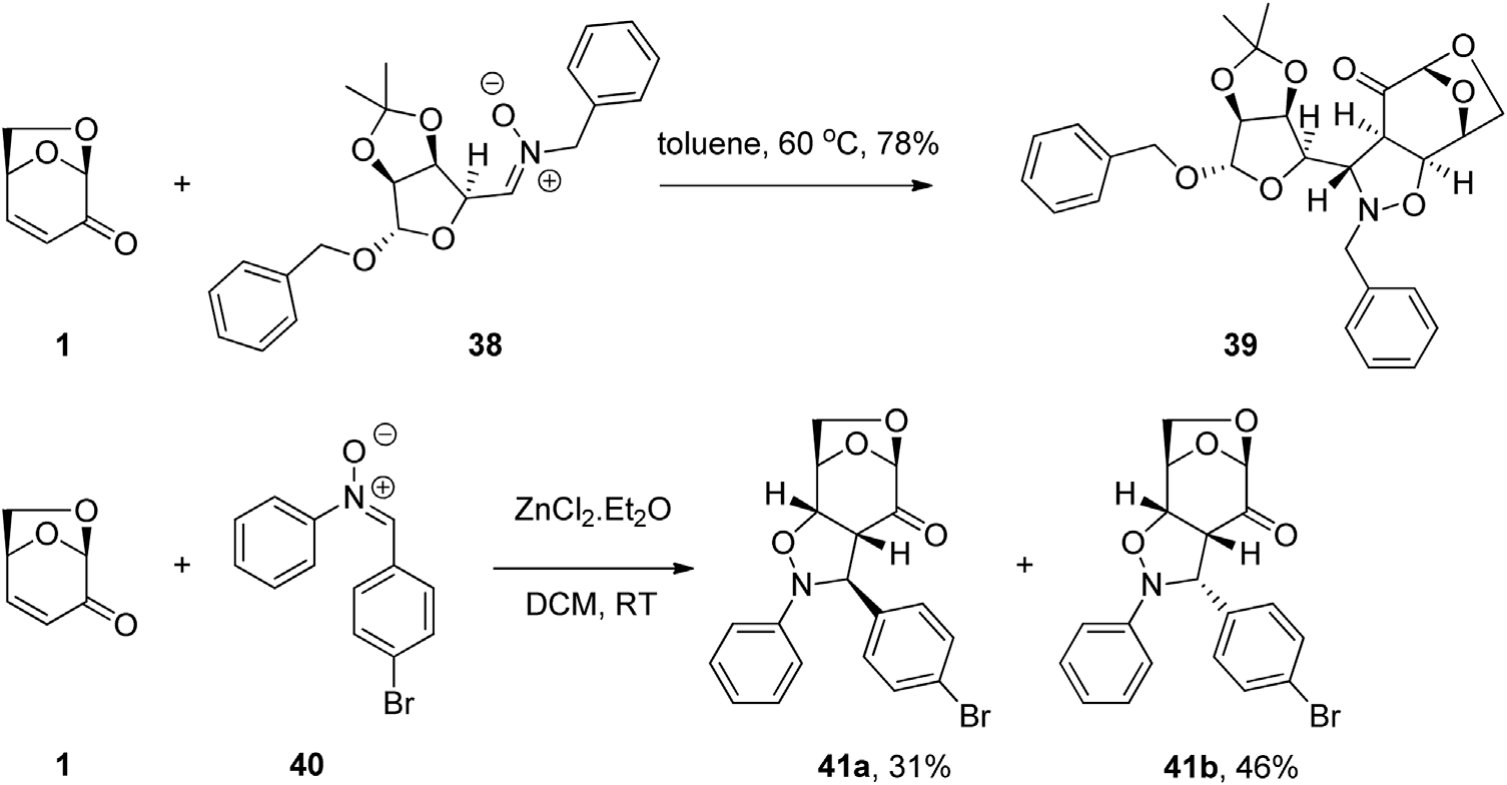
Cycloaddition reactions of LGO for the preparation of carbonic anhydrase and RAS activation inhibitors.

1,3-Dipolar cycloadditions of nitrones with LGO have also been used for the synthesis of biologically active derivatives. The selectivity of the process was controlled by the 1,6-anhydro bridge and the polarization of the conjugated alkene. Pratesi *et. al.* (2020) exploited the reactivity of the enone functionality of LGO in a dipolar cycloaddition reaction to give derivative 39 via reaction of LGO with nitrone 38 (Figure 12). One of the glycomimetics showed strong inhibition of the central nervous system expressed hCA VIII as well as selectivity towards a specific isoform, while inhibitory activity against tumor associated hCA IX (K_l_ = 35.9 nM) was shown by another of the LGO derivatives. A similar strategy was employed by Peri and co-workers for the construction of Ras activation inhibitors (Müller et al., 2009). Thus, the reaction of LGO with a series of aromatic nitrones 40 in the presence of zinc(II) chloride afforded the desired levoglucosenone fused isoxazolidines 41a and 41b as a mixture of diastereomers (Figure 12). These scaffolds were then further manipulated to give a total of nine functionalized derivatives and their biological activity examined. These molecules were shown to be a novel set of Ras inhibitors with interesting biological activity both *in vitro* and towards cells against a representative set of human cancer cell lines.

### Using LGO to Intercept Existing Routes to Pharmaceuticals

**FIGURE 13:**
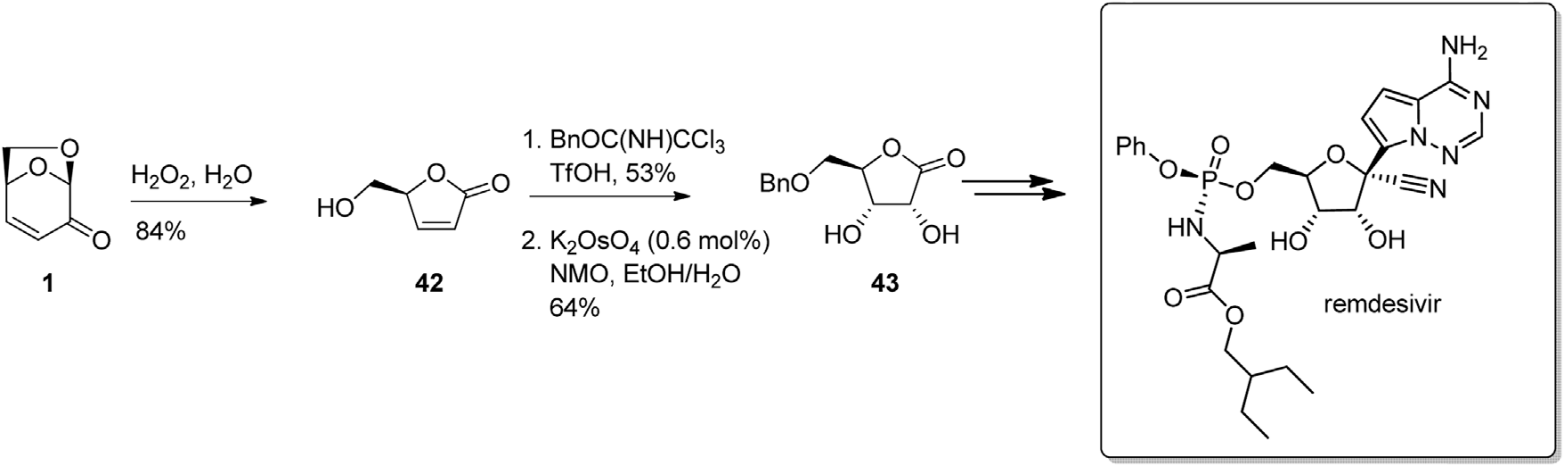
Conversion of LGO into protected ribonolactone derivatives used to synthesise antiviral compounds.

LGO and its derivatives are attractive building block for the synthesis of key intermediates used in the production of pharmaceuticals, largely due to its well-understood chemistry and availability on scale. As an example, Allais and his team at AgroParisTech have developed straight forward protocols for the facile conversion of LGO to 5-*O-*benzyl-D-(+)-ribo-1,4-lactone 43, which is a key building block for the synthesis of the COVID-19 anti-infective drug remdesivir (Figure 13) (Flourat et al., 2015; Moreaux et al., 2019). In this work, Baeyer-Villiger oxidation of LGO to the butenolide 42 followed by alcohol protection and *syn*-dihydroxylation gave the protected ribonolactone 43 in good yield and selectivity.

**FIGURE 14:**
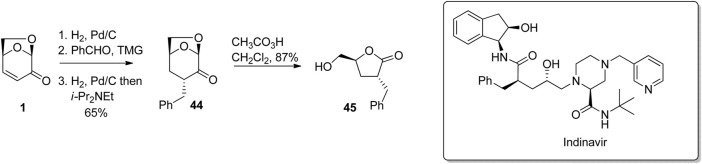
Preparation of a key intermediate that can be used in the preparation of indinavir.

The ready transformation of LGO or its derivatives to 5-hydroxymethylbutyrolactones via the Baeyer-Villiger reaction can be used to access a variety of pharmaceuticals. For example, a hydrogenation-aldol condensation-hydrogenation sequence starting with LGO afforded ketone 44, which was then hydrogenated and oxidized using peracetic acid to give the benzylated butyrolactone 45 (Figure 14) (Ledingham E. T. et al., 2017). The configuration of the benzyl group in the intermediate ketone 44 was controlled by steric interactions with the oxymethylene bridge, resulting in the requisite *anti*-configuration of substituents in the final butyrolactone. The original route to 45, which was used to construct the core section of the HIV protease inhibitor indinavir reported by Dorsey et al. (1994) was prepared in 5-steps from glutamic acid. This synthesis involved protection/deprotections steps, and strong base, while the more atom-economic synthesis of 45 starting with LGO proceeds in four-high yielding steps in 57% overall yield. This same benzylated butyrolactone has been used for the preparation of vasoactive intestinal peptide inhibitors, and drugs for Alzheimer’s disease.

## Conclusion

This brief outline has identified a diverse set of bioactive materials that can be prepared from LGO, and include materials that retain the chiral bicyclic structure, or transfer the chirality to new motifs. The orthogonal reactive groups, and the diastereoselectivity imparted by the bicyclic ring-system gives unique opportunities to prepare bioactive compounds. The trend towards using single enantiomer drugs in place of racemates places LGO in a unique position among renewable chemicals. LGO is one of the few chiral pyrolysis products obtained from cellulose, and is the principle product in bio oils formed from the acid-catalysed pyrolysis of cellulose. This sets it apart from other platform chemicals such as furfural, itaconic acid, and chloromethyl furfural which have lost the chirality present in the carbohydrate precursor, and require asymmetric strategies for their use in chiral bioactive synthesis. The development of reactions for the LGO ring-system coupled with its availability on large scale will provide additional opportunities to access known and new bioactive chemicals.

## References

[B1] Bamba M. Nishikawa T. Isobe M. (1996). Tin-assisted Cyclization for Chiral Cyclohexane Synthesis, an Alternative Route to (−)-tetrodotoxin Skeleton. Tetrahedron Lett. 37 (45), 8199–8202. 10.1016/0040-4039(96)01860-6

[B2] Banwell M. G. Liu X. Connal L. A. Gardiner M. G. (2020). Synthesis of Functionally and Stereochemically Diverse Polymers via Ring-Opening Metathesis Polymerization of Derivatives of the Biomass-Derived Platform Molecule Levoglucosenone Produced at Industrial Scale. Macromolecules 53 (13), 5308–5314. 10.1021/acs.macromol.0c01305

[B3] Bianchi C. Ceriotti G. (1975). Chemical and Pharmacological Investigations of Constituents of Eleutherine Bulbosa (Miller) Urb. (Iridaceae). J. Pharm. Sci. 64 (8), 1305–1308. 10.1002/jps.2600640809 807711

[B4] Bonneau G. Peru A. A. M. Flourat A. L. Allais F. (2018). Organic Solvent- and Catalyst-free Baeyer-Villiger Oxidation of Levoglucosenone and Dihydrolevoglucosenone (Cyrene): a Sustainable Route to (S)-γ-hydroxymethyl-α,β-butenolide and (S)-γ-hydroxymethyl-γ-butyrolactone. Green Chem. 20 (11), 2455–2458. 10.1039/c8gc00553b

[B5] Chew S. Ferrier R. J. Sinnwell V. (1988). An Approach to the Pyranonaphthoquinones. Carbohydr. Res. 174, 161–168. 10.1016/0008-6215(88)85089-4

[B6] Comba M. B. Suárez A. G. Sarotti A. M. Mangione M. I. Spanevello R. A. Giordano E. D. V. (2016). Synthesis of a 3-Thiomannoside. Org. Lett. 18 (8), 1748–1751. 10.1021/acs.orglett.6b00428 27053242

[B7] Comba M. B. Tsai Y.-h. Sarotti A. M. Mangione M. I. Suárez A. G. Spanevello R. A. (2018). Levoglucosenone and its New Applications: Valorization of Cellulose Residues. Eur. J. Org. Chem. 2018 (5), 590–604. 10.1002/ejoc.201701227

[B8] Czubatka-Bieńkowska A. Sarnik J. Macieja A. Galita G. Witczak Z. J. Poplawski T. (2017). Thio-functionalized Carbohydrate Thiosemicarbazones and Evaluation of Their Anticancer Activity. Bioorg. Med. Chem. Lett. 27 (12), 2713–2720. 10.1016/j.bmcl.2017.04.051 28506752

[B9] Debsharma T. Behrendt F. N. Laschewsky A. Schlaad H. (2019). Ring‐Opening Metathesis Polymerization of Biomass‐Derived Levoglucosenol. Angew. Chem. Int. Ed. 58 (20), 6718–6721. 10.1002/anie.201814501 30835937

[B10] Delbart D. I. Giri G. F. Cammarata A. Pan M. D. Bareño L. A. Amigo N. L. (2022). Antineoplastic Activity of Products Derived from Cellulose-Containing Materials: Levoglucosenone and Structurally-Related Derivatives as New Alternatives for Breast Cancer Treatment. Invest. New Drugs 40, 30–41. 10.1007/s10637-021-01167-6 34478029

[B11] Diot-Néant F. Rastoder E. Miller S. A. Allais F. (2018). Chemo-enzymatic Synthesis and Free Radical Polymerization of Renewable Acrylate Monomers from Cellulose-Based Lactones. ACS Sustain. Chem. Eng. 6 (12), 17284–17293. 10.1021/acssuschemeng.8b04707

[B12] Dorsey B. D. Levin R. B. McDaniel S. L. Vacca J. P. Guare J. P. Darke P. L. (1994). L-735,524: the Design of a Potent and Orally Bioavailable HIV Protease Inhibitor. J. Med. Chem. 37 (21), 3443–3451. 10.1021/jm00047a001 7932573

[B13] Eiden F. Denk F. Höfner G. (1994). ZNS-wirksame Pyrane: Amin- und arylsubstituierte Dioxabicyclooctane. Arch. Pharm. Pharm. Med. Chem. 327 (7), 405–412. 10.1002/ardp.19943270702 8074612

[B14] Essig M. G. (1986). Michael Additions of Thiols to Levoglucosenone. Carbohydr. Res. 156, 225–231. 10.1016/s0008-6215(00)90115-0

[B15] Fadlallah S. Peru A. A. M. Flourat A. L. Allais F. (2020). A Straightforward Access to Functionalizable Polymers through Ring-Opening Metathesis Polymerization of Levoglucosenone-Derived Monomers. Eur. Polym. J. 138, 109980. 10.1016/j.eurpolymj.2020.109980

[B16] Faizullina L. K. Safarov M. G. Spirikhin L. V. Kolosnitsyn V. S. Kondrova Y. A. Valeev F. A. (2011). Reaction of Nitroalkanes with Levoglucosenone and its α-bromo and α-iodo Derivatives. Cyclopentaannulation of α-halocyclenones. Russ. J. Org. Chem. 47 (6), 914–919. 10.1134/s1070428011060145

[B17] Flourat A. L. Peru A. A. M. Teixeira A. R. S. Brunissen F. Allais F. (2015). Chemo-enzymatic Synthesis of Key Intermediates (S)-γ-hydroxymethyl-α,β-butenolide and (S)-γ-hydroxymethyl-γ-butyrolactone via Lipase-Mediated Baeyer-Villiger Oxidation of Levoglucosenone. Green Chem. 17 (1), 404–412. 10.1039/c4gc01231c

[B18] Freskos J. N. Swenton J. S. (1985). Annelation Reaction of Levoglucosenone. Chiral Intermediates for the Synthesis of naphtho[2.3-C]pyran-5,10-Quinone Antibiotics. J. Chem. Soc. Chem. Commun. 10, 658–659. 10.1039/c39850000658

[B19] Giordano E. D. V. Frinchaboy A. Suárez A. G. Spanevello R. A. (2012). Synthesis of Tri-O-acetyl-d-allal from Levoglucosenone. Org. Lett. 14 (17), 4602–4605. 10.1021/ol302061a 22920651

[B20] Giri G. F. Danielli M. Marinelli R. A. Spanevello R. A. (2016). Cytotoxic Effect of Levoglucosenone and Related Derivatives against Human Hepatocarcinoma Cell Lines. Bioorg. Med. Chem. Lett. 26 (16), 3955–3957. 10.1016/j.bmcl.2016.07.007 27422336

[B21] Giri G. F. Viarengo G. Furlán R. L. E. Suárez A. G. Garcia Véscovi E. Spanevello R. A. (2017). Soybean Hulls, an Alternative Source of Bioactive Compounds: Combining Pyrolysis with Bioguided Fractionation. Industrial Crops Prod. 105, 113–123. 10.1016/j.indcrop.2017.05.005

[B22] Hughes L. McElroy C. R. Whitwood A. C. Hunt A. J. (2018). Development of Pharmaceutically Relevant Bio-Based Intermediates Though Aldol Condensation and Claisen-Schmidt Reactions of Dihydrolevoglucosenone (Cyrene). Green Chem. 20 (19), 4423–4427. 10.1039/c8gc01227j

[B23] Jæger Pedersen M. Pedersen C. M. (2021). Reactivity, Selectivity, and Synthesis of 4‐ C ‐Silylated Glycosyl Donors and 4‐Deoxy Analogues. Angew. Chem. 133 (5), 2721–2725. 10.1002/ange.202009209 33025650

[B24] Koseki K. Ebata T. Kawakami H. Matsushita H. Itoh K. Naoi Y. (1991). Method of Preparing (S)-γ-Hydroxymethyl-α, β-butenolide. European Patent No EP0411403A1. Munich, Germany: European Patent Office.

[B25] Koseki K. Ebata T. Kawakami H. Matsushita H. Naoi Y. Itoh K. (1990). A Method for Easy Preparation of Optically Pure (S)‐5‐Hydroxy‐2‐penten‐4‐olide and (S)‐5‐Hydroxypentan‐4‐olide. Heterocycles 31 (40), 423–426. 10.3987/com-89-5300

[B26] Kühlborn J. Groß J. Opatz T. (2020). Making Natural Products from Renewable Feedstocks: Back to the Roots? Nat. Prod. Rep. 37 (3), 380–424. 10.1039/c9np00040b 31625546

[B27] Lawrence C. H. Raverty W. D. Duncan A. J. (2012). Method for Converting Lignocellulosic Materials into Useful Chemicals. Washington, DC: U.S. Patent and Trademark Office. U.S. Patent Application US20120111714A1.

[B28] Ledingham E. Merritt C. Sumby C. Taylor M. Greatrex B. (2017a). Stereoselective Cyclopropanation of (-)-Levoglucosenone Derivatives Using Sulfonium and Sulfoxonium Ylides. Synthesis 49 (12), 2652–2662. 10.1055/s-0036-1588971

[B29] Ledingham E. T. Stockton K. P. Greatrex B. W. (2017b). Efficient Synthesis of an Indinavir Precursor from Biomass-Derived (-)-Levoglucosenone. Aust. J. Chem. 70 (10), 1146–1150. 10.1071/ch17227

[B30] Liu X. Carr P. Gardiner M. G. Banwell M. G. Elbanna A. H. Khalil Z. G. (2020). Levoglucosenone and its Pseudoenantiomer Iso-Levoglucosenone as Scaffolds for Drug Discovery and Development. ACS Omega 5 (23), 13926–13939. 10.1021/acsomega.0c01331 32566859 PMC7301580

[B31] Moreaux M. Bonneau G. Peru A. Brunissen F. Janvier M. Haudrechy A. (2019). High-Yielding Diastereoselective Syn -Dihydroxylation of Protected HBO: An Access to D-(+)-Ribono-1,4-lactone and 5-O -Protected Analogues. Eur. J. Org. Chem. 2019 (7), 1600–1604. 10.1002/ejoc.201801780

[B32] Sarotti A. M. Zanardi M. Spanevello R. A. Suarez A. G. (2012a). Recent Applications of Levoglucosenone as Chiral Synthon. Cos 9 (4), 439–459. 10.2174/157017912802651401

[B33] Müller C. Gomez-Zurita Frau M. A. Ballinari D. Colombo S. Bitto A. Martegani E. (2009). Design, Synthesis, and Biological Evaluation of Levoglucosenone-Derived Ras Activation Inhibitors. ChemMedChem 4 (4), 524–528. 10.1002/cmdc.200800416 19226500

[B34] Nagai K. Kiguchi S. Koyama H. Hume W. E. Tsujimoto S. (2015). Method for Producing 4′-ethynyl d4T. Geneva, Switzerland: WIPO. PCT Application WO/2009/084655.

[B35] Nishikawa T. Isobe M. (2013). Synthesis of Tetrodotoxin, a Classic but Still Fascinating Natural Product. Chem. Rec. 13 (3), 286–302. 10.1002/tcr.201200025 23661608

[B36] Novikov R. A. Rafikov R. R. Shulishov E. V. Konyushkin L. D. Semenov V. V. Tomilov Y. V. (2009). Reactions of Levoglucosenone and its Derivatives with Diazo Compounds. Russ. Chem. Bull. 58 (2), 327–334. 10.1007/s11172-010-0011-9

[B37] Pratesi D. Matassini C. Goti A. Angeli A. Carta F. Supuran C. T. (2020). Glycomimetic Based Approach toward Selective Carbonic Anhydrase Inhibitors. ACS Med. Chem. Lett. 11 (5), 727–731. 10.1021/acsmedchemlett.9b00590 32435377 PMC7236246

[B38] Samet A. V. Niyazymbetov M. E. Semenov V. V. Laikhter A. L. Evans D. H. (1996). Comparative Studies of Cathodically-Promoted and Base-Catalyzed Michael Addition Reactions of Levoglucosenone. J. Org. Chem. 61 (25), 8786–8791. 10.1021/jo961019g 11667855

[B39] Samet A. V. Shestopalov A. M. Lutov D. N. Rodinovskaya L. A. Shestopalov A. A. Semenov V. V. (2007). Preparation of Chiral Cyclopropanes with a Carbohydrate Fragment from Levoglucosenone. Tetrahedron Asymmetry 18 (16), 1986–1989. 10.1016/j.tetasy.2007.08.013

[B40] Samet А. V. Lutov D. N. Firgang S. I. Lyssenko K. A. Semenov V. V. (2011). A Concise Approach to Chiral Chromenes Based on Levoglucosenone. Tetrahedron Lett. 52 (23), 3026–3028. 10.1016/j.tetlet.2011.04.004

[B41] Sarotti A. M. Spanevello R. A. Suárez A. G. Echeverría G. A. Piro O. E. (2012b). 1,3-Dipolar Cycloaddition Reactions of Azomethine Ylides with a Cellulose-Derived Chiral Enone. A Novel Route for Organocatalysts Development. Org. Lett. 14 (10), 2556–2559. 10.1021/ol3008588 22545814

[B42] Shafizadeh F. Chin P. P. S. (1977). Preparation of 1,6-Anhydro-3,4-Dideoxy-β-D-Glycero-Hex-3-Enopyranos-2-Ulose (Levoglucosenone) and Some Derivatives Thereof. Carbohydr. Res. 58 (1), 79–87. 10.1016/s0008-6215(00)83406-0

[B43] Shafizadeh F. Furneaux R. H. Stevenson T. T. (1979). Some Reactions of Levoglucosenone. Carbohydr. Res. 71 (1), 169–191. 10.1016/s0008-6215(00)86069-3

[B44] Shafizadeh F. Ward D. D. Pang D. (1982). Michael-addition Reactions of Levoglucosenone. Carbohydr. Res. 102 (1), 217–230. 10.1016/s0008-6215(00)88064-7

[B45] Sharipov B. T. Davidova A. N. Ryabova A. S. Galimzyanova N. F. Valeev F. A. (2019). Synthesis and Fungicidal Activity of Methylsulfanylmethyl Ether Derivatives of Levoglucosenone. Chem. Heterocycl. Comp. 55 (1), 31–37. 10.1007/s10593-019-02415-7

[B46] Smith R. A. Raugi D. N. Wu V. H. Leong S. S. Parker K. M. Oakes M. K. (2015). The Nucleoside Analog BMS-986001 Shows Greater *In Vitro* Activity against HIV-2 Than against HIV-1. Antimicrob. Agents Chemother. 59 (12), 7437–7446. 10.1128/aac.01326-15 26392486 PMC4649195

[B47] Stockton K. P. Greatrex B. W. (2016). Synthesis of Enantiopure Cyclopropyl Esters from (−)-levoglucosenone. Org. Biomol. Chem. 14 (31), 7520–7528. 10.1039/c6ob00933f 27424764

[B48] Swenton J. S. Freskos J. N. Dalidowicz P. Kerns M. L. (1996). A Facile Entry into Naphthopyran Quinones via an Annelation Reaction of Levoglucosenone. The Total Synthesis of (−)-Hongconin1. J. Org. Chem. 61 (2), 459–464. 10.1021/jo951607e 11666961

[B49] Tagirov A. R. Biktagirov I. M. Galimova Y. S. Faizullina L. K. Salikhov S. M. Valeev F. A. (2015). Opening of the 1,6-anhydro Bridge with Selective Reduction of the Acetal Moiety in Levoglucosenone and its Derivatives. Russ. J. Org. Chem. 51 (4), 569–575. 10.1134/s1070428015040181

[B50] Teixeira A. R. S. Flourat A. L. Peru A. A. M. Brunissen F. Allais F. (2016). Lipase-Catalyzed Baeyer-Villiger Oxidation of Cellulose-Derived Levoglucosenone into (S)-γ-Hydroxymethyl-α,β-Butenolide: Optimization by Response Surface Methodology. Front. Chem. 4, 16. 10.3389/fchem.2016.00016 27148523 PMC4835721

[B51] Tolstikov A. G. Tolstikov G. A. (2007). Unsaturated Sugars in Enantiospecific Synthesis of Natural Low-Molecular Bioregulators and Their Structural Analogues. Russ. J. Bioorg Chem. 33 (1), 3–23. 10.1134/s1068162007010025 17375656

[B52] Trahanovsky W. S. Ochaoda J. M. Wang C. Revell K. D. Arvidson K. B. Wang Y. (2003). “A Convenient Procedure for the Preparation of Levoglucosenone and its Conversion to Novel Chiral Derivatives,” in Carbohydrate Synthons in Natural Products Chemistry: Synthesis, Functionalization, and Applications. Editors Witczak Z. J. Tatsuta K. (Washington, DC: American Chemical Society).

[B53] Tsai Y.-h. Borini Etichetti C. M. Cicetti S. Girardini J. E. Spanevello R. A. Suárez A. G. (2020). Design, Synthesis and Evaluation of Novel Levoglucosenone Derivatives as Promising Anticancer Agents. Bioorg. Med. Chem. Lett. 30 (14), 127247. 10.1016/j.bmcl.2020.127247 32527547

[B54] Tsai Y.-h. Borini Etichetti C. M. Di Benedetto C. Girardini J. E. Martins F. T. Spanevello R. A. (2018). Synthesis of Triazole Derivatives of Levoglucosenone as Promising Anticancer Agents: Effective Exploration of the Chemical Space through Retro-Aza-Michael//aza-Michael Isomerizations. J. Org. Chem. 83 (7), 3516–3528. 10.1021/acs.joc.7b03141 29481076

[B55] Tsypysheva I. P. Valeev F. A. Vasil'eva E. V. Spirikhin L. V. Tolstikov G. A. (2000). Stereochemical Differentiation in the Reactions of Organometallic Reagents with Levoglucosenone and Some of its Dihydro Derivatives. Russ. Chem. Bull. 49 (7), 1237–1240. 10.1007/bf02495766

[B56] Urabe D. Nishikawa T. Isobe M. (2006). An Efficient Total Synthesis of Optically Active Tetrodotoxin from Levoglucosenone. Chem. Asian J. 1 (1‐2), 125–135. 10.1002/asia.200600038 17441047

[B57] Valeev F. A. Gorobets E. V. Miftakhov M. S. (1999). Reactions of 3-iodolevoglucosenone with Sodium Derivatives of Some CH Acids. Chiral Cyclopropanes and Stable Oxetenes. Russ. Chem. Bull. 48 (1), 152–156. 10.1007/bf02494418

[B58] Ward D. D. Shafizadeh F. (1981). Bromination of Levoglucosenone. Carbohydr. Res. 93 (2), 284–287. 10.1016/s0008-6215(00)80858-7

[B59] Westman J. Wiman K. Mohell N. (2007). Levoglucosenone Derivatives for the Treatment of Disorders Such as Cancer, Autoimmune Diseases and Heart Diseases. Geneva, Switzerland: WIPO. PCT Application WO/2007/139497.

[B60] Witczak Z. J. (2007). “New Stereoselective Functionalization of Cellulose-Derived Pyrolysis Derivatives: Levoglucosenone and its Dimer,” in Materials, Chemicals, and Energy from Forest Biomass. Editor Argyropoulos D. S. (American Chemical Society), 332–349. 10.1021/bk-2007-0954.ch021

[B61] Witczak Z. J. Sarnik J. Czubatka A. Forma E. Poplawski T. (2014). Thio-sugar Motif of Functional CARB-Pharmacophore for Antineoplastic Activity. Part 2. Bioorg. Med. Chem. Lett. 24 (24), 5606–5611. 10.1016/j.bmcl.2014.10.095 25466184

[B62] Witczak Z. J. Tatsuta K. (2002). Carbohydrate Synthons in Natural Products Chemistry: Synthesis, Functionalization, and Applications. Washington, DC: American Chemical Society.

[B63] Yatsynich E. A. Petrov D. V. Valeev F. A. Dokichev V. A. (2003). Synthesis of Pyrazolines Based on Levoglucosenone. Chem. Nat. Compd. 39 (4), 337–339. 10.1023/b:conc.0000003411.19962.39

